# Unique Insights in the Cervicovaginal *Lactobacillus iners* and *L*. *crispatus* Proteomes and Their Associations with Microbiota Dysbiosis

**DOI:** 10.1371/journal.pone.0150767

**Published:** 2016-03-10

**Authors:** Hanneke Borgdorff, Stuart D. Armstrong, Hanne L. P. Tytgat, Dong Xia, Gilles F. Ndayisaba, Jonathan M. Wastling, Janneke H. H. M. van de Wijgert

**Affiliations:** 1 Amsterdam Institute for Global Health and Development (AIGHD) and Department of Global Health, Academic Medical Center, Amsterdam, The Netherlands; 2 Institute of Infection and Global Health, University of Liverpool, Liverpool, United Kingdom; 3 Laboratory of Microbiology, Wageningen University, Wageningen, The Netherlands; 4 Centre of Microbial and Plant Genetics, Catholic University Leuven, Leuven, Belgium; 5 Laboratory of Environmental Ecology and Applied Microbiology, University of Antwerp, Antwerp, Belgium; 6 Rinda Ubuzima, Kigali, Rwanda; 7 Faculty of Natural Sciences, Keele University, Keele, United Kingdom; Fred Hutchinson Cancer Center, UNITED STATES

## Abstract

**Background:**

A *Lactobacillus*-dominated cervicovaginal microbiota (VMB) protects women from adverse reproductive health outcomes, but the role of *L*. *iners* in the VMB is poorly understood. Our aim was to explore the association between the cervicovaginal *L*. *iners* and *L*. *crispatus* proteomes and VMB composition.

**Methods:**

The vaginal proteomes of 50 Rwandan women at high HIV risk, grouped into four VMB groups (based on 16S rDNA microarray results), were investigated by mass spectrometry using cervicovaginal lavage (CVL) samples. Only samples with positive 16S results for *L*. *iners* and/or *L*. *crispatus* within each group were included in subsequent comparative protein analyses: *Lactobacillus crispatus*-dominated VMB cluster (with 16S-proven *L*. *iners* (n_i_) = 0, and with 16S-proven *L*. *crispatus* (n_c_) = 5), *L*. *iners*-dominated VMB cluster (n_i_ = 11, n_c_ = 4), moderate dysbiosis (n_i_ = 12, n_c_ = 2); and severe dysbiosis (n_i_ = 8, n_c_ = 2). The relative abundances of proteins that were considered specific for *L*. *iners* and *L*. *crispatus* were compared among VMB groups.

**Results:**

Forty *Lactobacillus* proteins were identified of which 7 were specific for *L*. *iners* and 11 for *L*. *crispatus*. The relative abundances of *L*. *iners* DNA starvation/stationary phase protection protein (DPS), and the glycolysis enzymes glyceraldehyde-3-phosphate dehydrogenase (GAPDH) and glucose-6-phosphate isomerase (GPI), were significantly decreased in women with *L*. *iners*-containing dysbiosis compared to women with a *L*. *iners*-dominated VMB, independent of vaginal pH and *L*. *iners* abundance. Furthermore, *L*. *iners* DPS, GAPDH, GPI, and fructose-bisphosphate aldolase (ALDO) were significantly negatively associated with vaginal pH. Glycolysis enzymes of *L*. *crispatus* showed a similar negative, but nonsignificant, trend related to dysbiosis.

**Conclusions:**

Most identified *Lactobacillus* proteins had conserved intracellular functions, but their high abundance in CVL supernatant might imply an additional extracellular (moonlighting) role. Our findings suggest that these proteins can be important in maintaining a *Lactobacillus*-dominated VMB. Functional studies are needed to investigate their roles in vaginal bacterial communities and whether they can be used to prevent vaginal dysbiosis.

## Introduction

A *Lactobacillus*-dominated cervicovaginal microbiota (VMB) is generally considered healthy, and is associated with low bacterial diversity and low vaginal pH [1]. VMB dysbiosis, defined by high bacterial diversity and presence of a mixture of (facultative) anaerobic bacteria, is associated with adverse reproductive outcomes, including increased HIV risk [2], and increased risk of preterm birth in pregnant women [3]. The golden standard of detecting dysbiosis has long been the diagnosis of bacterial vaginosis (BV), a clinical syndrome diagnosed by microscopy of Gram-stained vaginal smears (Nugent scoring [4]), or wet mount microscopy and clinical criteria (Amsel criteria [5]). Molecular methods have improved the resolution of VMB dysbiosis detection in research settings: it is now possible to distinguish between *Lactobacillus* species, and identify subtypes of dysbiosis [1]. Despite these advances in characterising the VMB, it still remains unknown how dysbiosis develops and how it can be treated effectively and sustainably [6].

The most prevalent vaginal lactobacilli are *Lactobacillus crispatus* and *L*. *iners*. A *L*. *crispatus*-dominated VMB is considered more beneficial than a *L*. *iners*-dominated VMB, because it is less likely to shift to dysbiosis and is associated with lower prevalence of sexually transmitted infections (STIs) [1, 7, 8]. Additionally, in contrast to other vaginal lactobacilli, *L*. *iners* is frequently found in dysbiotic VMB compositions and its beneficial role has therefore been debated. However, one could argue that the tolerance of *L*. *iners* to a changing VMB and vaginal pH also reflects its adaptation to the vaginal niche and *L*. *iners* may be the pioneer bacterium to shift a dysbiotic VMB to a *Lactobacillus*-dominated VMB. Its ability to adapt to a changing VMB has been emphasised by a study comparing the vaginal metatranscriptome between women with and without dysbiosis that found differential expression of 10% of *L*. *iners* genes [9]. The authors hypothesised that *L*. *iners* is able to adapt to dysbiosis by modifying its gene expression of metabolism, cytolysis and antibacteriophage defense genes. However, no studies have investigated the association between the VMB and the *Lactobacillus* proteome.

In this study, we compared the relative abundance of *L*. *crispatus* and *L*. *iners* proteins in cervicovaginal lavages (CVLs) among 50 Rwandan women with different VMB compositions. Our aim was to explore how *L*. *crispatus* and *L*. *iners* adapt to or influence the VMB, and may influence the risk of adverse outcomes.

## Materials and Methods

### Study design

For this study, stored samples from the Kigali HIV incidence study (KHIS) were analysed. The KHIS study estimated the HIV prevalence and incidence in Rwandan female sex workers (n = 800) between 2006 and 2009 [10]. The study was approved by the National Ethics Committee, Rwanda, and the Columbia University Medical Center Review Board, USA. All participants provided written informed consent. Briefly, after a screening survey, a selection of participants (397 HIV negative and 141 HIV-positive participants) was followed for two years at regular intervals. During screening and follow-up visits, women were interviewed about sociodemographics and sexual risk behaviour, and tested for HIV, pregnancy, (bacterial and viral) STIs, BV, and vaginal yeasts [10, 11]. Women received treatment for curable STIs, symptomatic BV and candidiasis at the study clinic, and were referred to other local clinics for care related to HIV, pregnancy, and abnormal cervical cytology. Women also received HIV counselling and condoms free of charge.

Previously, the VMB compositions of KHIS participants were characterised and clustered cross-sectionally using a phylogenetic microarray [7]. Subsequently, a selection was made of 50 participants based on their VMB composition as described below, for further cervicovaginal human (as described in [12]) and bacterial proteomics analyses (this study) of CVLs taken during the same pelvic exam. Selected women were 18–45 years, not pregnant, and their CVLs were not macroscopically bloody. S1 Table contains the sociodemographic, behavioural, and clinical characteristics of these 50 women, as also described previously [12].

### Sample collection

During pelvic examination, the vaginal pH was measured by pressing a pH paper strip against the vaginal wall (pH range 2–9 with 0.5 increments). CVLs of 5 mL were collected as described previously [12] and centrifuged at 1,000 x g for 10 min within 4 h of collection. Because we were interested in the interaction of *L*. *iners* and *L*. *crispatus* with their environment we analysed the supernatant (which contains mainly extracellular proteins) instead of the pellet (which contains mainly intracellular proteins). Supernatants were filtered using a sterile 0.2 mm cellulose acetate membrane (VWS International, Lutterworth, UK) and stored at -80°C prior to testing. Cervical samples were collected using cervical spatulas and cytobrushes, and were stored at -80°C in Preservcyt medium (ThinPrep Pap Test; Cytyc Corporation, Boxborough, MA) until microbiota analyses.

### Selection of participants

Previously, cervicovaginal microbiota analysis was performed using a phylogenetic microarray (TNO, Zeist, the Netherlands [7]). Briefly, semi-quantitative abundance of vaginal bacteria was determined by quantifying 16S rDNA amplicon hybridization to the phylogenetic microarray, and was expressed as normalized signal/background (S/B) ratios [7]. We refer to this semi-quantitative abundance as “abundance” throughout this manuscript. The normalized S/B ratio of 251 probes that returned consistent results were used for unsupervised clustering analyses [13]. The resulting clusters were categorised into four groups as described previously [7, 12]: *Lactobacillus crispatus*-dominated VMB (group 1), *L*. *iners*-dominated VMB (group 2), moderate dysbiosis (group 3), and severe dysbiosis (group 4). Groups 1 and 2 were characterised by low bacterial diversity and low abundance of dysbiosis-associated bacteria. Most women from group 1 had a *L*. *crispatus*-dominated microbiota, and women from group 2 had a *L*. *iners*-dominated microbiota or co-dominance of *L*. *iners* and *G*. *vaginalis*. Women from groups 3 and 4 had mixed microbiota with high abundance of *G*. *vaginalis*, *Prevotella* spp., and *Atopobium vaginae*. However, bacterial richness and diversity were lower in group 3 compared to group 4, and group 3 women had lower prevalence of STIs [7]. Therefore, we refer to group 3 as ‘moderate dysbiosis’ and group 4 as ‘severe dysbiosis’.

For this study, 50 participants from the VMB groups were selected as follows: seven women from the *L*. *crispatus*-dominated cluster, 11 women from the *L*. *iners*-dominated cluster who had low abundance of *G*. *vaginalis* (S/B ratio < 5), 14 women from the moderately dysbiotic group, and 18 women from the severely dysbiotic group. All selected women in the *L*. *crispatus*-dominated and *L*. *iners*-dominated clusters were BV-negative by Nugent scoring, all women in the severely dysbiotic group were BV-positive, and women in the moderately dysbiotic group had mixed BV diagnoses (S1 Table; [12]).

### Protein extraction and mass spectrometry

Protein extraction and mass spectrometry were performed as described previously [12]. Briefly, total protein concentrations were determined using the Pierce Coomassie Plus (Bradford) Protein Assay (Thermo Scientific, Rockford, IL). Sample protein content and volume were normalized with 25 mM ammonium bicarbonate. Mass spectrometry was performed using the nanoACQUITY-nLC system (Waters) coupled to an LTQ-Orbitrap Velos (ThermoFisher Scientific, Bremen, Germany) mass spectrometer. Runs were time aligned using default settings of Progenesis LC—MS (version 4.1, Nonlinear Dynamics, Newcastle, UK) and using an auto selected run as reference. Peaks were picked by the software and filtered to include only peaks with a charge state between +2 and +7. Peptide intensities were normalized against the reference run. Throughout this paper, these normalized total ion intensities are referred to as ‘relative abundance’.

Peptide identification was performed using Mascot search engine (version 2.3.02, Matrix Science, London, UK). Tandem mass spectrometry data were searched against three databases: a human proteins database (UniProt reviewed, version February 2014, containing 20,276 sequences) and two micro-organism protein databases that we compiled ourselves as follows: one database containing all NCBI reference sequences (*L*. *iners*, *L*. *crispatus*, *L*. *jensenii*, *L*. *gasseri*, *L*. *vaginalis* and ‘*Lactobacillus* multispecies’; compiled on 14 January 2016), and one database containing sequences of 23 non-*Lactobacillus* vaginal bacterial species (based on [1]), *Trichomonas vaginalis*, *Candida albicans*, and *Candida glabrata* (reference sequences where possible; compiled on 26–28 March 2013; see S2 Table for the complete list). The human database and the bacterial database containing non-*Lactobacillus* strains were included to provide quality control for the identification of *Lactobacillus* proteins; when a protein matched a *Lactobacillus* protein, but not any protein in the other two databases, we could be more certain about the *Lactobacillus* origin of the protein. Search parameters were as follows: precursor mass tolerance 10 ppm, fragment mass tolerance 0.6 Da, false discovery rate <1%, and individual ion scores >13 (indicating identity or extensive homology at p<0.05). Only bacterial proteins for which at least one unique peptide and at least two peptides overall (including non-unique peptides) were identified were included in further analyses, as has been done in previous cervicovaginal studies [14–16]. Furthermore, *L*. *iners* or *L*. *crispatus* proteins for which only one unique peptide was identified were checked for consistent differential abundance among VMB groups (Progenesis ANOVA p-value <0.05), and proteins were only included if a series of at least 4 continuous fragment ions were observed after manual inspection of individual MS/MS spectra. The mass spectrometry proteomics data have been deposited in the PRIDE partner repository of the ProteomeXchange Consortium with the dataset identifiers PXD003176 and 10.6019/PXD003176 [17].

### Data analyses

Statistical analyses were performed using STATA (release 12, StataCorp, College Station, TX, USA) and R (release 3.1.3, R Foundation for Statistical Computing, Vienna, Austria).

In view of redundancy and homology in the *Lactobacillus* peptide databases, we applied additional criteria based on 16S rDNA microarray data to ensure species specificity of *Lactobacillus* proteins. These criteria were based on the assumption that proteins with high relative abundance in samples with only one highly abundant *Lactobacillus* sp. originated from that *Lactobacillus* species. Taken together, the strict criteria to classify proteins as *L*. *iners* proteins were: 1) the best match in the Mascot search was a *L*. *iners* or ‘*Lactobacillus* multispecies’ protein, 2) the mean ion intensity in women with a *L*. *iners*-dominated VMB was at least 2-fold higher, and the median ion intensity was equal to or higher than in women with no *L*. *iners* as determined by 16S rDNA microarray (i.e. women with a *L*. *crispatus*-dominated VMB and women with VMB dysbiosis without *L*. *iners*). Similar criteria were used for classification of *L*. *crispatus* proteins, with the difference that women without *L*. *crispatus* were used as a reference group (i.e. women with a *L*. *iners*-dominated VMB or women with VMB dysbiosis without *L*. *crispatus*). Proteins initially identified as ‘*Lactobacillus* multispecies’ proteins were thus used in the classification of both *L*. *iners* and *L*. *crispatus* proteins, but could only be classified as a protein from one out of both *Lactobacillus* species.

Only samples with positive 16S results for *L*. *iners* and/or *L*. *crispatus* within each group were included in subsequent comparative *L*. *iners/L*. *crispatus* protein analyses. The *L*. *crispatus*-dominated VMB cluster (n = 7) included 5 women with positive 16S rDNA microarray results for *L*. *crispatus* and 2 samples that had negative results for *L*. *crispatus*- and *L*. *iners* 16S rDNA (but had *L*. *jensenii* or *L*. *gasseri*, and similarly low bacterial diversity and low abundance of dysbiosis-associated bacteria to be clustered together with women with a *L*. *crispatus*-dominated VMB [13]). In the *L*. *iners*-dominated cluster (n = 11), 7 women had positive results for *L*. *iners* and 4 positive results for *L*. *iners* and *L*. *crispatus*. In the moderately dysbiotic cluster (n = 14), 10 women had positive results for *L*. *iners*, 2 for *L*. *iners* and *L*. *crispatus*, and 2 tested negative for *Lactobacillus* 16S rDNA. In the severely dysbiotic cluster (n = 18), 7 women had positive results for *L*. *iners*, 1 for *L*. *crispatus*, 1 for *L*. *iners* and *L*. *crispatus*, and 9 tested negative for lactobacilli.

Relative protein abundance for each identified *L*. *iners* and *L*. *crispatus* protein was compared among VMB groups and among vaginal pH categories, using two-sided Mann-Whitney pairwise tests. We used three vaginal pH categories (4–5, 5–6, and ≥6, respectively) in the *L*. *iners* protein analyses, but only two (4–5 and ≥5, respectively) in the *L*. *crispatus* protein analyses due to the small sample sizes. Analyses of *L*. *iners* proteins were also stratified for *L*. *iners* abundance (using two categories: below or above the median S/B ratio) to differentiate between changes in *L*. *iners* protein abundance due to changes in *L*. *iners* abundance or for other reasons. For *L*. *iners* proteins that were statistically significantly associated with dysbiosis in bivariable analyses, multivariable linear regression models adjusted for *L*. *iners* abundance (log-transformed microarray S/B ratio) and vaginal pH categories were fitted.

## Results

### Classification of identified *Lactobacillus* proteins

Using mass spectrometry, 549 human proteins [12] and 40 *Lactobacillus* proteins were identified in 50 CVLs. Of the 40 *Lactobacillus* proteins, 18 were matched to *L*. *crispatus*, 14 to *L*. *iners*, and 8 to *Lactobacillus ‘multispecies’*. Seven of the 22 *L*. *iners/L*. *‘multispecies’* proteins passed the strict criteria (see Materials and Methods section) and were therefore classified as specific *L*. *iners* proteins (Table 1). Of these, 4 were identified by ≥2 unique peptides: glyceraldehyde-3-phosphate dehydrogenases (GAPDH_1, a GAPDH homologue matching multiple *L*. *iners* strains in the NCBI database), DNA starvation/stationary phase protection protein (DPS), pyruvate kinase (PK), and elongation factor Tu (EFtu). *L*. *iners* GAPDH_2 (a GAPDH homologue matching *L*. *kefiri* and *L*. *parakefiri* in the NCBI database), fructose-bisphosphate aldolase (ALDO), and glucose-6-phosphate isomerase (GPI), were identified with a unique peptide count of 1.

**Table 1:** Cervicovaginal *Lactobacillus iners* and *L*. *crispatus* proteins identified by mass spectrometry.

Protein	Accession	Peptide count	Unique peptides	Confidence score	Abbreviation given
*L*. *iners proteins*					
glyceraldehyde-3-phosphate dehydrogenase	WP_006729586.1	12	10	1329.11	GAPDH_1
DNA starvation/stationary phase protection protein	WP_006730124.1	2	2	163.87	DPS
elongation factor Tu	WP_006730011.1	2	2	132.69	EFtu
pyruvate kinase	WP_006729992.1	2	2	82.89	PK
glyceraldehyde-3-phosphate dehydrogenase	WP_054769853.1	5	1	401.22	GAPDH_2
fructose-bisphosphate aldolase	WP_006728980.1	4	1	391.8	ALDO
glucose-6-phosphate isomerase	WP_006736984.1	2	1	176.28	GPI
*L*. *crispatus proteins*					
glyceraldehyde-3-phosphate dehydrogenase	WP_005718691.1	19	13	2016.42	GAPDH
enolase	WP_005727546.1	5	5	257.67	ENO
fructose-bisphosphate aldolase	WP_005725669.1	4	2	158.61	ALDO_1
fructose-bisphosphate aldolase	WP_005728047.1	5	2	517.02	ALDO_2
pullulanase type I	WP_005728646.1	3	3	359.31	PulA
cell division protein	WP_035450284.1	4	4	201.83	CDP
hydrolase	WP_035163526.1	2	2	171.35	HL
YSIRK signal domain/LPXTG anchor domain surface protein	WP_043884492.1	5	5	611.13	SD
hypothetical protein	WP_005725426.1	2	2	302.63	HP_1
hypothetical protein	WP_005718484.1	2	2	53.58	HP_2
glucose-6-phosphate isomerase	WP_023488057.1	2	1	102.07	GPI

All specific *L*. *iners* and *L*. *crispatus* proteins identified in cervicovaginal lavages of 50 women with different cervicovaginal microbiota compositions. Classification was based on Mascot search results (reference databases in S2 Table) and consistency with 16S rDNA microarray results, as described in the Materials and Methods section. Homologous proteins from different strains within the same species are numbered.

Eleven of the 26 *L*. *crispatus/L*. *‘multispecies’* proteins passed the criteria to be classified as specific *L*. *crispatus* proteins (Table 1). Glyceraldehyde-3-phosphate dehydrogenase (GAPDH), enolase (ENO), two fructose-bisphosphate aldolases (ALDO_1, ALDO_2, homologues matching different *L*. *crispatus* strains), pullulanase, type I (PulA), cell division protein (CDP), hydrolase (HL), YSIRK signal domain/LPXTG anchor domain surface protein (SD), and two hypothetical proteins (HP_1, HP_2) were identified by ≥2 unique proteins. Glucose-6-phosphate isomerases (GPI) was identified with a unique peptide count of 1.

All *L*. *iners/L*. *crispatus/L*. *‘multispecies’* proteins that were not classified as specific *L*. *iners* or *L*. *crispatus* proteins were considered nonspecific at species level because they were not more abundant in women with a *L*. *iners* or *L*. *crispatus*-dominant VMB by 16S rDNA microarray compared to women without *L*. *iners* or *L*. *crispatus* by 16S rDNA microarray, respectively.

### Bivariable and multivariable associations between *L*. *iners* proteins, dysbiosis and vaginal pH

**Fig 1:**
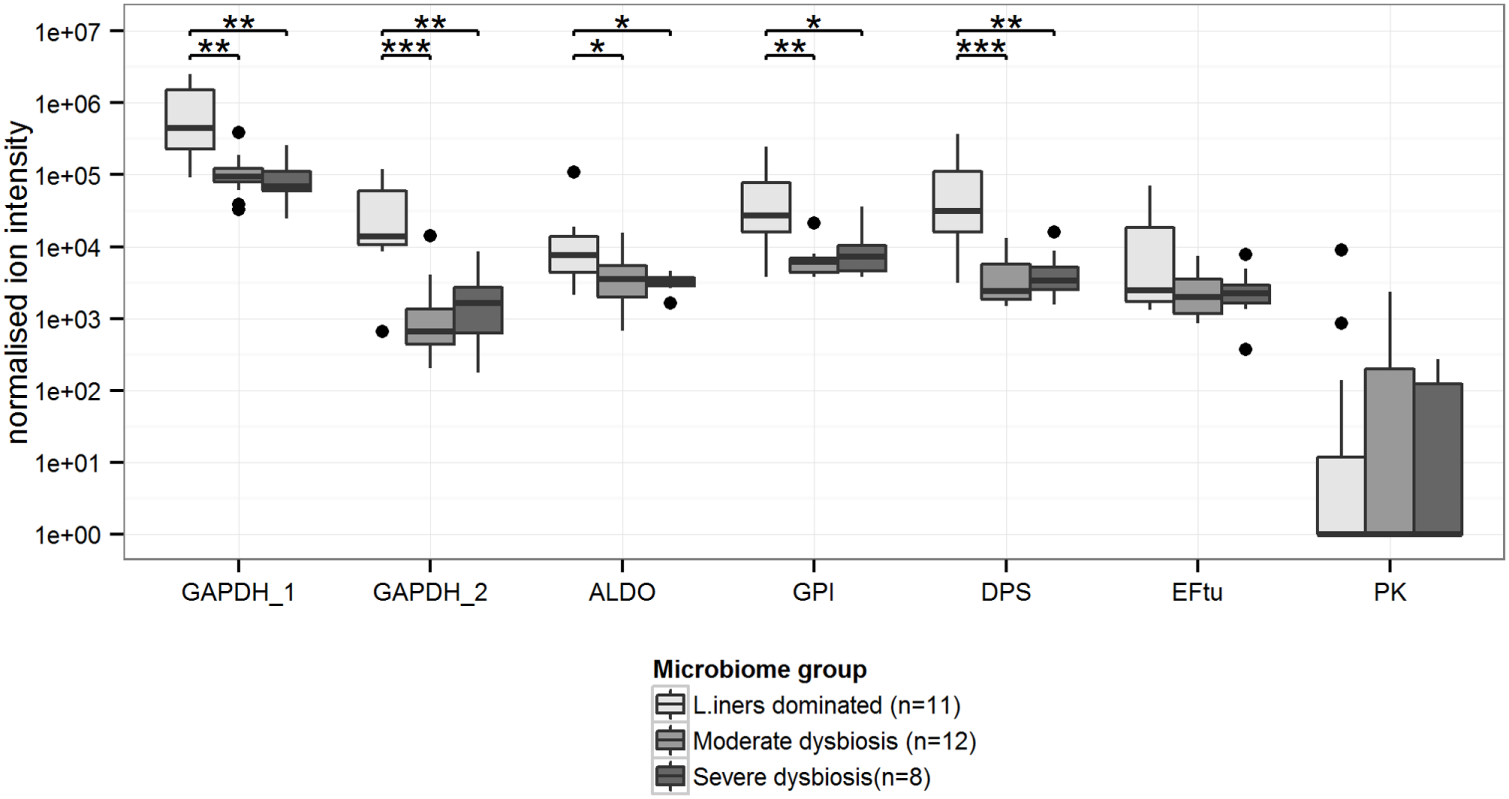
Relative abundances of *L*. *iners* proteins in cervicovaginal lavages of women with positive *L*. *iners* 16S rDNA microarray results (n = 31) among three cervicovaginal microbiota groups. GAPDH_1, GAPDH_2, ALDO, GPI, and DPS were significantly decreased in women with moderate and severe dysbiosis, compared to women with a *L*. *iners*-dominant cervicovaginal microbiota. Box plots represent median (black line), first and third quartiles (box) and range within 1.5 times the interquartile range from the box (whiskers). Outliers are plotted as points. *p-value<0.05; ** p-value<0.01; ***p-value<0.001. Abbreviations: GAPDH: glyceraldehyde-3-phosphate dehydrogenase; ALDO: fructose-bisphosphate aldolase; GPI: glucose-6-phosphate isomerase; DPS: DNA starvation/stationary phase protection protein; EFtu: elongation factor Tu; PK: pyruvate kinase.

**Fig 2:**
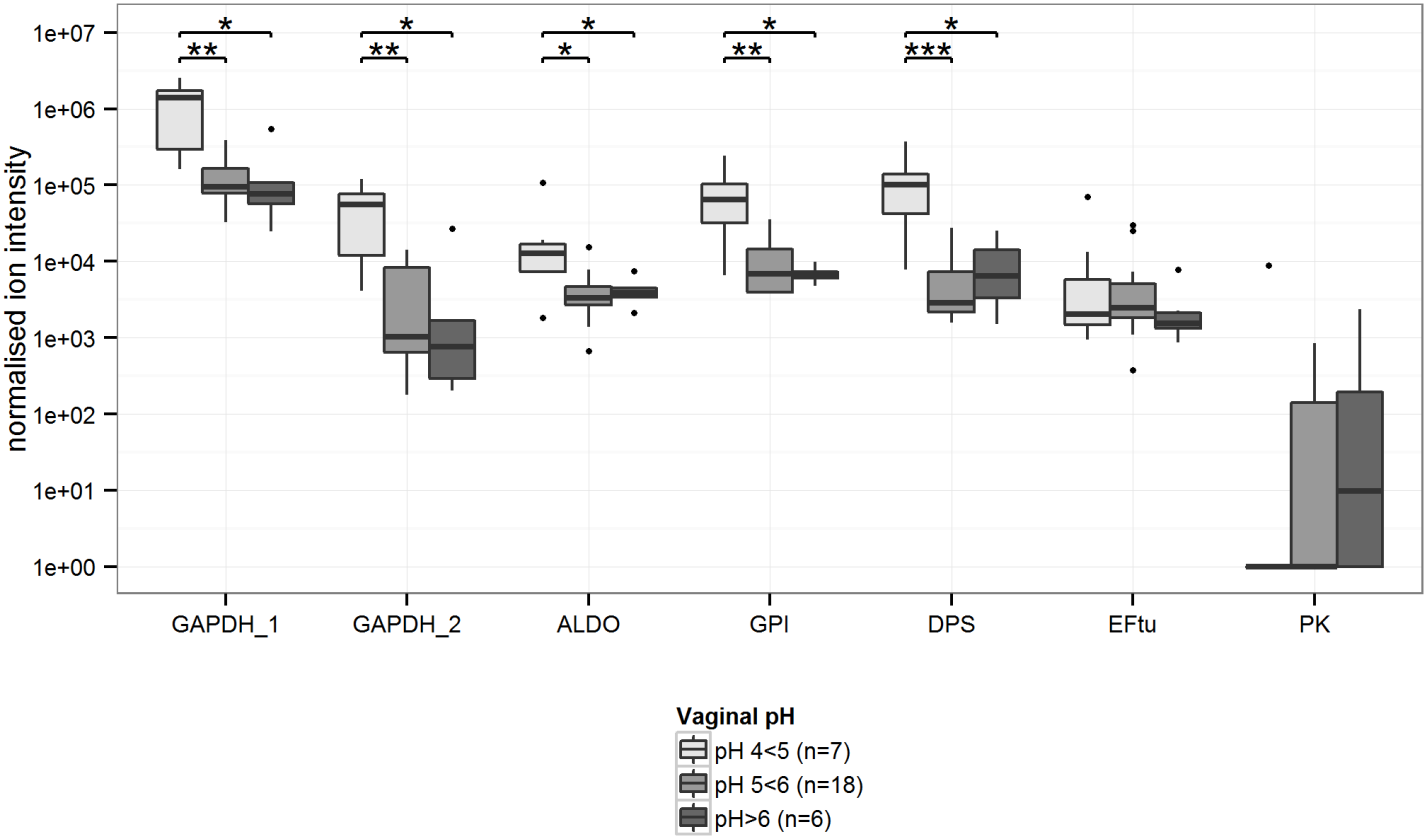
Relative abundances of *L*. *iners* proteins in cervicovaginal lavages of women with positive *L*. *iners* 16S rDNA microarray results (n = 31) among three vaginal pH categories. GAPDH_1, GAPDH_2, ALDO, GPI, and DPS were significantly decreased in women with a vaginal pH ≥5, compared to women with a vaginal pH between 4 and 5. Box plots represent median (black line), first and third quartiles (box) and range within 1.5 times the interquartile range from the box (whiskers). Outliers are plotted as points. *p-value<0.05; ** p-value<0.01; ***p-value<0.001. Abbreviations: GAPDH: glyceraldehyde-3-phosphate dehydrogenase; ALDO: fructose-bisphosphate aldolase; GPI: glucose-6-phosphate isomerase; DPS: DNA starvation/stationary phase protection protein; EFtu: elongation factor Tu; PK: pyruvate kinase.

GAPDH_1 was the most highly abundant *L*. *iners* protein in the CVLs, followed by ALDO, GPI, and DPS (Fig 1). The relative abundance of GAPDH_1, GAPDH_2, GPI, ALDO, and DPS was significantly higher in women with a *L*. *iners*-dominated VMB than in women with moderate or severe dysbiosis (Fig 1). The negative association between GAPDH_1, GAPDH_2, GPI, and DPS and moderate dysbiosis, and GAPDH_2 and DPS and severe dysbiosis, remained statistically significant when limiting the analysis to women with high abundance of *L*. *iners* (S/B ratio >70, n = 16; S1 Fig). The median vaginal pH of participants was 5, with a range of 4–7. The relative abundance of GADPH_1, GAPDH_2, GPI, ALDO, and DPS was negatively associated with vaginal pH categories (Fig 2). These associations remained statistically significant when limiting the analysis to women with a *L*. *iners*-dominated VMB (S2 Fig; n = 11). In multivariable analyses, moderate dysbiosis was independently associated with decreased relative abundance of GAPDH_1, GAPDH_2, GPI, and DPS, and severe dysbiosis with decreased relative abundance of GAPDH_1, GAPDH_2, and DPS (Table 2). Furthermore, increasing vaginal pH was independently associated with decreasing relative abundance of GAPDH_1, GAPDH_2, ALDO, GPI, ALDO, and DPS.

**Table 2:** Multivariable linear regression analyses of the association between *L*. *iners* relative protein abundance, microbiota composition, vaginal pH, and *L*. *iners* abundance.

		Dependent variables									
		GAPDH_1^1^		GAPDH_2^1^		ALDO^1^		GPI^1^		DPS^1^	
Independent variables	n	beta	95% CI	beta	95% CI	beta	95% CI	beta	95% CI	beta	95% CI
Microbiota group											
*L*. *iners* dominated	11	ref		ref		ref		ref		ref	
Moderate dysbiosis	12	-0.44	(-0.78,-0.10)	-0.89	(-1.38, -0.40)	-0.26	(-0.61,0.09)	-0.42	(-0.76,-0.09)	-0.61	(-0.91,-0.29)
Severe dysbiosis	8	-0.47	(-0.86,-0.08)	-0.61	(-1.16, -0.05)	-0.26	(-0.66,0.13)	-0.24	(-0.62,0.13)	-0.52	(-0.87,-0.17)
Vaginal pH											
4–5	7	ref		ref		ref		ref		ref	
5–6	18	-0.49	(-0.87,-0.11)	-0.61	(-1.15, -0.07)	-0.45	(-0.83,-0.07)	-0.59	(-0.96,-0.23)	-0.80	(-1.14,-0.46)
≥6	6	-0.56	(-1.03,-0.10)	-0.79	(-1.45, -0.13)	-0.37	(-0.84,0.10)	-0.68	(-1.13,-0.23)	-0.54	(-0.96,-0.13)
S/B ratio *L*. *iners*^1^		0.24	(-0.23,0.71)	0.81	(0.15, 1.48)	-0.26	(-0.73,0.22)	0.04	(-0.41,0.49)	0.56	(0.13,0.98)

The residuals of the linear regression model were normally distributed as tested using the Shapiro-Wilk test.

^1^Log10-transformed values. Abbreviations: S/B: signal/background ratio, CI: confidence interval, GAPDH: glyceraldehyde-3-phosphate dehydrogenase; ALDO: fructose-bisphosphate aldolase; GPI: glucose-6-phosphate isomerase; DPS: DNA starvation/stationary phase protection protein.

### Association between *L*. *crispatus* proteins, dysbiosis and pH

**Fig 3:**
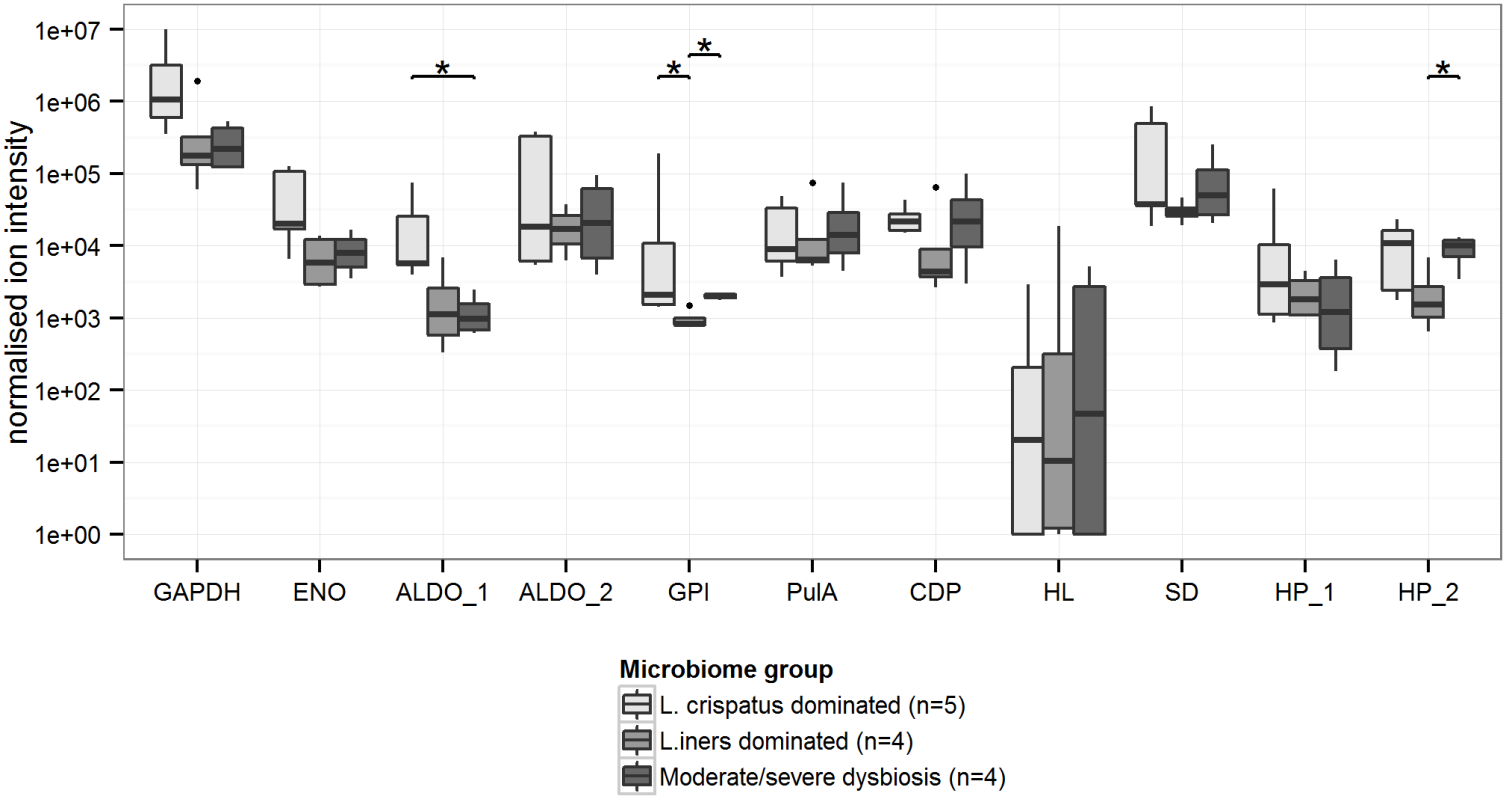
Relative abundances of *L*. *crispatus* proteins in cervicovaginal lavages of women with positive *L*. *crispatus* 16S rDNA microarray results (n = 13) among three cervicovaginal microbiota groups. Homologous proteins from different strains are numbered (Table 1). Box plots represent median (black line), first and third quartiles (box) and range within 1.5 times the interquartile range from the box (whiskers). Outliers are plotted as points. *p-value<0.05. Abbreviations: GAPDH: glyceraldehyde-3-phosphate dehydrogenase; ENO: enolase; ALDO: fructose-bisphosphate aldolase; GPI: glucose-6-phosphate isomerase; PulA: pullulanase type I; CDP: cell division protein; HL: hydrolase; SD: YSIRK signal domain/LPXTG anchor domain surface protein; HP: hypothetical protein.

**Fig 4:**
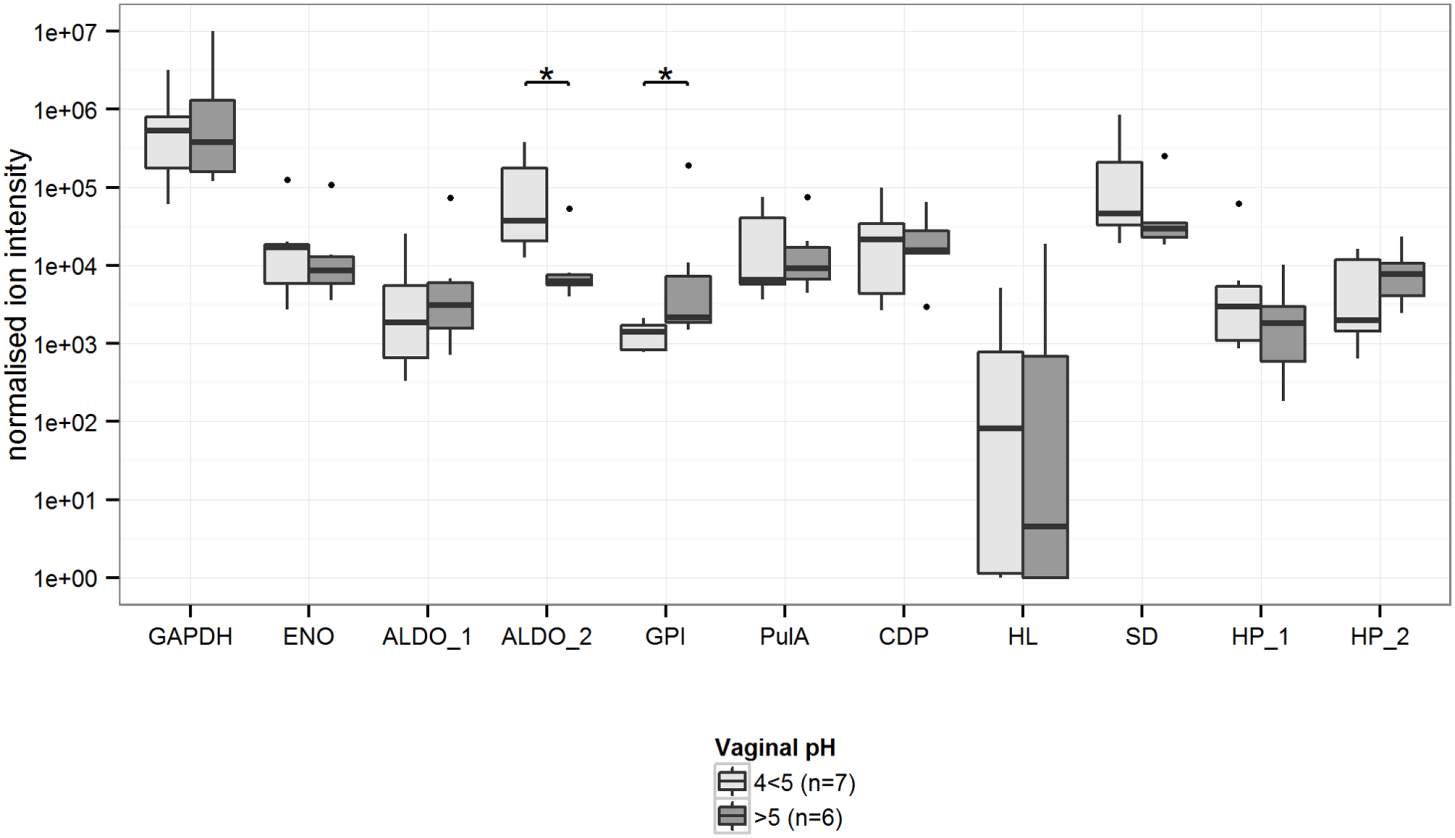
Relative abundances of *L*. *crispatus* proteins in cervicovaginal lavages of women with positive *L*. *crispatus* 16S rDNA microarray results (n = 13) among two vaginal pH categories. Homologous proteins from different strains are numbered (Table 1). Box plots represent median (black line), first and third quartiles (box) and range within 1.5 times the interquartile range from the box (whiskers). Outliers are plotted as points. *p-value<0.05. Abbreviations: GAPDH: glyceraldehyde-3-phosphate dehydrogenase; ENO: enolase; ALDO: fructose-bisphosphate aldolase; GPI: glucose-6-phosphate isomerase; PulA: pullulanase type I; CDP: cell division protein; HL: hydrolase; SD: YSIRK signal domain/LPXTG anchor domain surface protein; HP: hypothetical protein.

Of the 11 specific *L*. *crispatus* proteins, only ALDO_1 was significantly decreased in severe dysbiosis (Fig 3). However, the glycolysis proteins GAPDH and ENO also showed a lower relative abundance in women with dysbiosis compared to in women with a *L*. *crispatus*-dominated VMB, but this was not statistically significant. ALDO_2 was not associated with dysbiosis, but was negatively associated with vaginal pH (Fig 4), and GPI was positively associated with vaginal pH. However, no overall trends between *L*. *crispatus* proteins and vaginal pH could be discerned.

## Discussion

We identified 4 specific *L*. *iners* and 10 specific *L*. *crispatus* proteins with ≥2 unique peptides in CVL supernatants of Rwandan women at high HIV risk. Of these, *L*. *iners* GAPDH_1 and DPS were negatively associated with VMB dysbiosis, independent of *L*. *iners* abundance and vaginal pH. Similar results were found for *L*. *iners* GAPDH_2 and GPI, which were identified with a single unique peptide. As expected, few women with dysbiosis had detectable levels of *L*. *crispatus* by 16S rDNA microarray [7], but a trend towards a similar negative association between *L*. *crispatus* glycolysis proteins and dysbiosis could be discerned.

Knowledge on expression and/or secretion of *L*. *iners* and *L*. *crispatus* proteins is scarce [9, 18–22]. GAPDH, ENO, and GPI were previously identified in the *L*. *crispatus* ST1 exoproteome *in vitro* [19, 21]. Furthermore, genomics and metatranscriptomics analyses predicted high expression of all *L*. *iners* proteins that we identified in this study in women with and without BV [9, 23]. The metatranscriptomics study reported no differential expression of *L*. *iners* glycolysis genes in women with and without BV, but did show a non-significant decrease in *L*. *iners* DPS (previously annotated as non-heme containing ferritin) expression in women with BV [9]. However, the study included only 4 women and may not be representative for women with different VMB compositions and different *L*. *iners* strains.

Most of the *L*. *iners* and *L*. *crispatus* proteins identified in this study are proteins with highly conserved intracellular functions. Their presence in CVL supernatant may be the result of either lysis of *Lactobacillus* spp. during collection or handling of the CVLs, or their pre-existent abundance in the *Lactobacillus* exoproteome, where they may have additional functions. In case of the former, the increased relative abundance of glycolysis enzymes could reflect an increase in *Lactobacillus* metabolic activity in VMB communities that are dominated by these lactobacilli. However, their presence in the *Lactobacillus* exoproteome is more likely for the following reasons. Firstly, dilution of lactobacilli in physiological saline (CVL procedure) causes no to minimal cell lysis, and cell lysis during or after centrifugation has not affected our results due to separate storage of supernatants from cell pellets. Secondly, high abundance of the glycolysis enzymes GAPDH, ENO, and GPI in the *L*. *crispatus* exoproteome is supported by previous *in vitro* studies [19, 21].

Many proteins with conserved intracellular functions are known to have additional extracellular functions in bacteria, including (competitive) adhesion, plasminogen binding, and immune modulation, called moonlighting (as reviewed in [24–26]). Although the presence of these moonlighting proteins is a relatively new concept, for all *Lactobacillus* glycolysis proteins identified in this study, additional extracellular functions have been described in closely related bacterial species (GAPDH, ALDO, GPI, PK and ENO; as reviewed in [25]).

*L*. *iners* GAPDH_1, GAPDH_2, GPI, ALDO, and DPS were significantly decreased in women with dysbiosis. For the glycolysis enzymes GAPDH, GPI, and ALDO, *in vitro* studies have provided compelling evidence of additional extracellular functions in related species including adhesion to the bacterial cell wall, mucin, plasminogen, and/or epithelial cells [19,21,27–29]. Furthermore, GAPDH has been found to be involved in competitive exclusion of pathogens *in vitro* [30] and immunomodulation in mice [31]. DPS has been shown to protect bacteria in *in vitro* studies, including *Streptococcus* spp., from iron overload and H_2_O_2_ [32,33], and to be involved in biofilm formation and tolerance against bacteriophages in *E*. *coli* [34]. However, more research is needed to clarify the role of ferritin-like proteins in lactobacilli. Based on these and our data, we hypothesise that increased abundance of glycolysis enzymes and DPS in the *Lactobacillus* exoproteome might benefit the survival, persistence, and resilience of lactobacilli. However, other potential explanations for our observation of increased abundance of these proteins in a *Lactobacillus*-dominated VMB are the presence of different *L*. *iners* strains in different VMB states, strain-independent adaptation of protein regulation in different VMB states, and/or a dysbiosis-associated increase in protein breakdown due to an increased concentration of bacterial and human proteases. For example, the secretion of *L*. *iners* moonlighting proteins might be downregulated during dysbiosis as a survival strategy to retain valuable enzymes intracellularly. However, this might lead to a reduction in *L*. *iners* fitness as its chances of outcompeting other bacteria and re-establishing a *Lactobacillus*-dominated VMB are curtailed.

The present study shows that *in vivo*, *L*. *iners* GAPDH_1 and DPS, and at lower quantification confidence also GAPDH_2, GPI, and ALDO, were significantly decreased at a vaginal pH range associated with dysbiosis (>4.5 [5]). This might reflect a decline in metabolic activity of *L*. *iners* with a subsequent decrease of lactic acid production (causing a pH elevation) and insufficient energy production to perform energy-consuming activities such as the secretion of proteins. Alternatively, these *L*. *iners* proteins may have detached from the bacterial cell wall during the pH increase associated with the transition to dysbiosis, as was described *in vitro* for *L*. *crispatus* GAPDH and GPI [20,21], and might have been diluted or washed away with vaginal secretions.

The number of women with detectable *L*. *crispatus* was low, especially in the dysbiotic groups. Therefore, in the analysis of *L*. *crispatus* proteins, no adjustments for *L*. *crispatus* abundance or pH could be performed. However, relative abundance of ALDO_1 was significantly lower in women with dysbiosis than women with a *L*. *crispatus*-dominated VMB, and the glycolysis proteins GAPDH and ENO also showed lower, but non-significant, relative abundance in women with dysbiosis. The function of these proteins and the adaptation to dysbiosis may thus be similar as in *L*. *iners*. However, many epidemiological studies have shown that *L*. *crispatus* is, in contrast to *L*. *iners*, strongly negatively associated with dysbiosis (as reviewed in [1]). We may not have found a strong association between *L*. *crispatus* proteins, dysbiosis, and vaginal pH in this study because of *L*. *crispatus*’ failure to adapt to dysbiosis. However, studies with a larger group of women with *L*. *crispatus*-containing dysbiosis are needed to investigate this further.

As mentioned above, sample sizes were small, especially for the *L*. *crispatus* analyses. Also, the concentration of *Lactobacillus* proteins was relatively low compared to the concentration of human proteins [12] in the samples. Due to detection limitations related to the dynamic range of the mass spectrometer, it is likely that only the most abundant *Lactobacillus* proteins were detected. Furthermore, as in all proteomics studies, we were dependent on the completeness and accuracy of publicly available protein annotation databases for our reference list of proteins. For example, we suspect that GAPDH_2 is erroneously mapped to *L*. *kefiri/L*. *parakefiri* instead of *L*. *iners*, because its relative abundance correlated to *L*. *iners* abundance and to GAPDH_1 (which mapped to *L*. *iners* strains only). Furthermore, *L*. *kefiri* and *L*. *parakefiri* have not been described to occur in the vaginal niche. However, we set to minimise the risk of misidentifying a protein lacking correct annotation as a *Lactobacillus* protein by applying additional criteria for protein identification based on microbiota composition by 16S rDNA microarray (Materials and Methods). Lastly, due to sample loading normalization, we report relative abundance instead of absolute concentration, but total protein concentrations in CVLs were not associated with VMB composition (data not shown).

Our *Lactobacillus* proteome analyses identified proteins that are likely to be involved in survival strategies of *L*. *iners* and *L*. *crispatus* in *Lactobacillus*-dominant and dysbiotic VMB compositions. The association between the relative abundance of potential *L*. *iners* moonlighting proteins and dominance of *L*. *iners*, independent of *L*. *iners* abundance, implies their importance in establishing and maintaining a *L*. *iners*-dominated microbiome. We recommend that future studies further investigate the function and secretion of these proteins by different vaginal *Lactobacillus* strains. Strains with high expression and/or secretion of these proteins could perhaps be used as vaginal probiotics to prevent vaginal dysbiosis and dysbiosis-associated adverse reproductive health outcomes.

## Supporting Information

S1 Fig Relative abundances of *L*. *iners proteins in cervicovaginal lavages of* women with high *L*. *iners* abundance (signal/background ratio >70, n = 16) among three cervicovaginal microbiota groups. GAPDH_1, GAPDH_2, GPI, and DPS were significantly decreased in women with dysbiosis, compared to women with a *L*. *iners*-dominant cervicovaginal microbiota. Box plots represent median (black line), first and third quartiles (box) and range within 1.5 times the interquartile range from the box (whiskers). Outliers are plotted as points. *p-value<0.05; ** p-value<0.01. Abbreviations: GAPDH: glyceraldehyde-3-phosphate dehydrogenase; ALDO: fructose-bisphosphate aldolase; GPI: glucose-6-phosphate isomerase; DPS: DNA starvation/stationary phase protection protein; EFtu: elongation factor Tu; PK: pyruvate kinase.
(TIFF)

S2 Fig Relative abundances of *L*. *iners* proteins in cervicovaginal lavages of women with a *L*. *iners*-dominated cervicovaginal microbiota (n = 11) among three vaginal pH categories. GAPDH_1, GAPDH_2, ALDO, GPI, and DPS were significantly decreased in women with a vaginal pH ≥5, compared to women with a vaginal pH between 4 and 5. Box plots represent median (black line), first and third quartiles (box) and range within 1.5 times the interquartile range from the box (whiskers). Outliers are plotted as points. *p-value<0.05. Abbreviations: GAPDH: glyceraldehyde-3-phosphate dehydrogenase; ALDO: fructose-bisphosphate aldolase; GPI: glucose-6-phosphate isomerase; DPS: DNA starvation/stationary phase protection protein; EFtu: elongation factor Tu; PK: pyruvate kinase.
(TIFF)

S1 Table Sociodemographic, behavioural, and clinical characteristics of 50 women from different microbiota groups. (XLSX)

S2 Table Reference databases used for peptide identification by Mascot search engine. (XLSX)
