# Supplementary figures and images for: Unique Insights in the Cervicovaginal Lactobacillus iners and L. crispatus Proteomes and Their Associations with Microbiota Dysbiosis
Source: PLoS One. 2016 Mar 10;11(3):e0150767. doi: 10.1371/journal.pone.0150767 (PMC4786256; doi:10.1371/journal.pone.0150767)

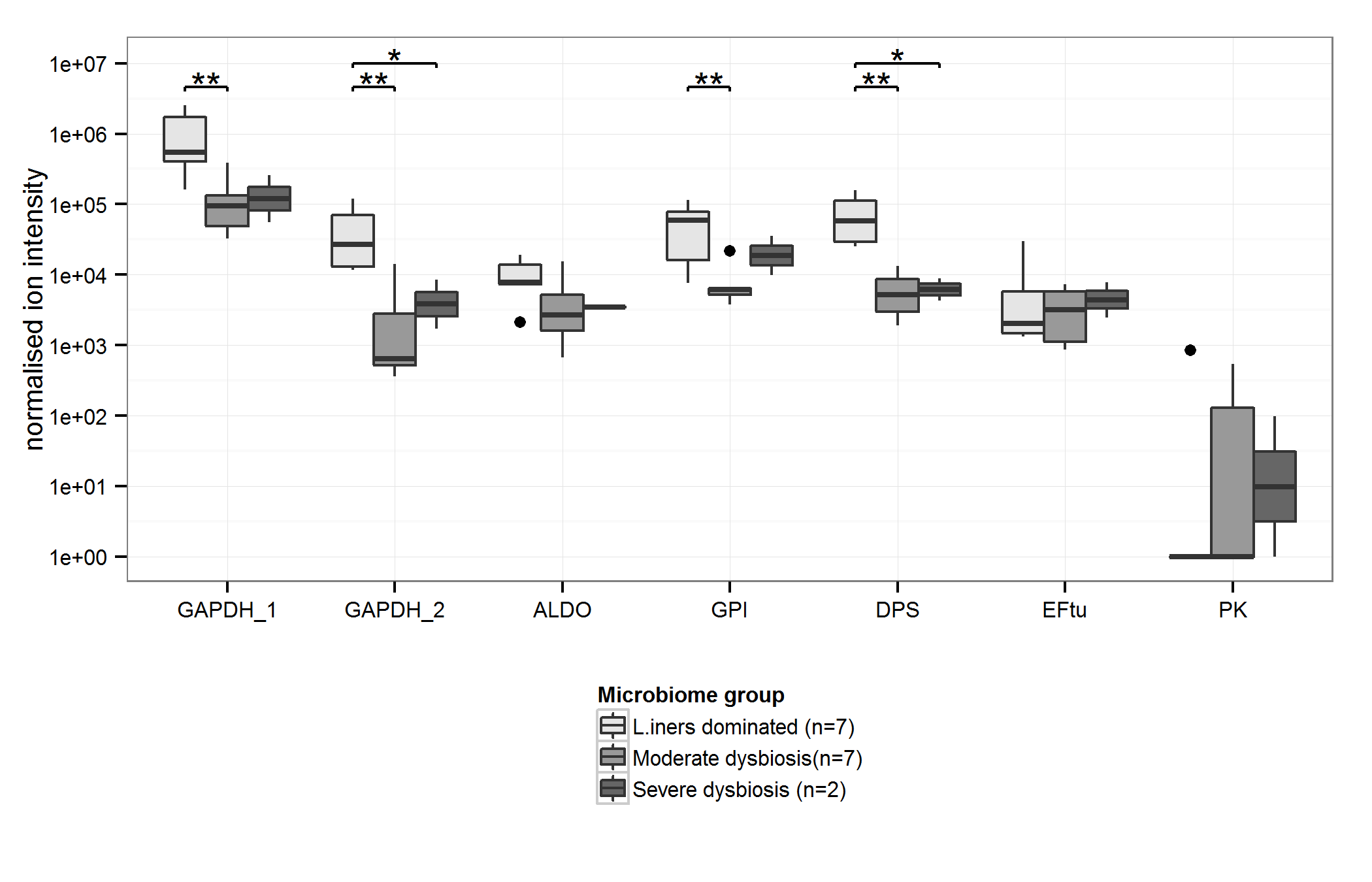

Supplement: S1 Fig — GAPDH_1, GAPDH_2, GPI, and DPS were significantly decreased in women with dysbiosis, compared to women with a L. iners-dominant cervicovaginal microbiota. Box plots represent median (black line), first and third quartiles (box) and range within 1.5 times the interquartile range from the box (whiskers). Outliers are plotted as points. *p-value<0.05; ** p-value<0.01. Abbreviations: GAPDH: glyceraldehyde-3-phosphate dehydrogenase; ALDO: fructose-bisphosphate aldolase; GPI: glucose-6-phosphate isomerase; DPS: DNA starvation/stationary phase protection protein; EFtu: elongation factor Tu; PK: pyruvate kinase. (TIFF) [file pone.0150767.s001.tiff]

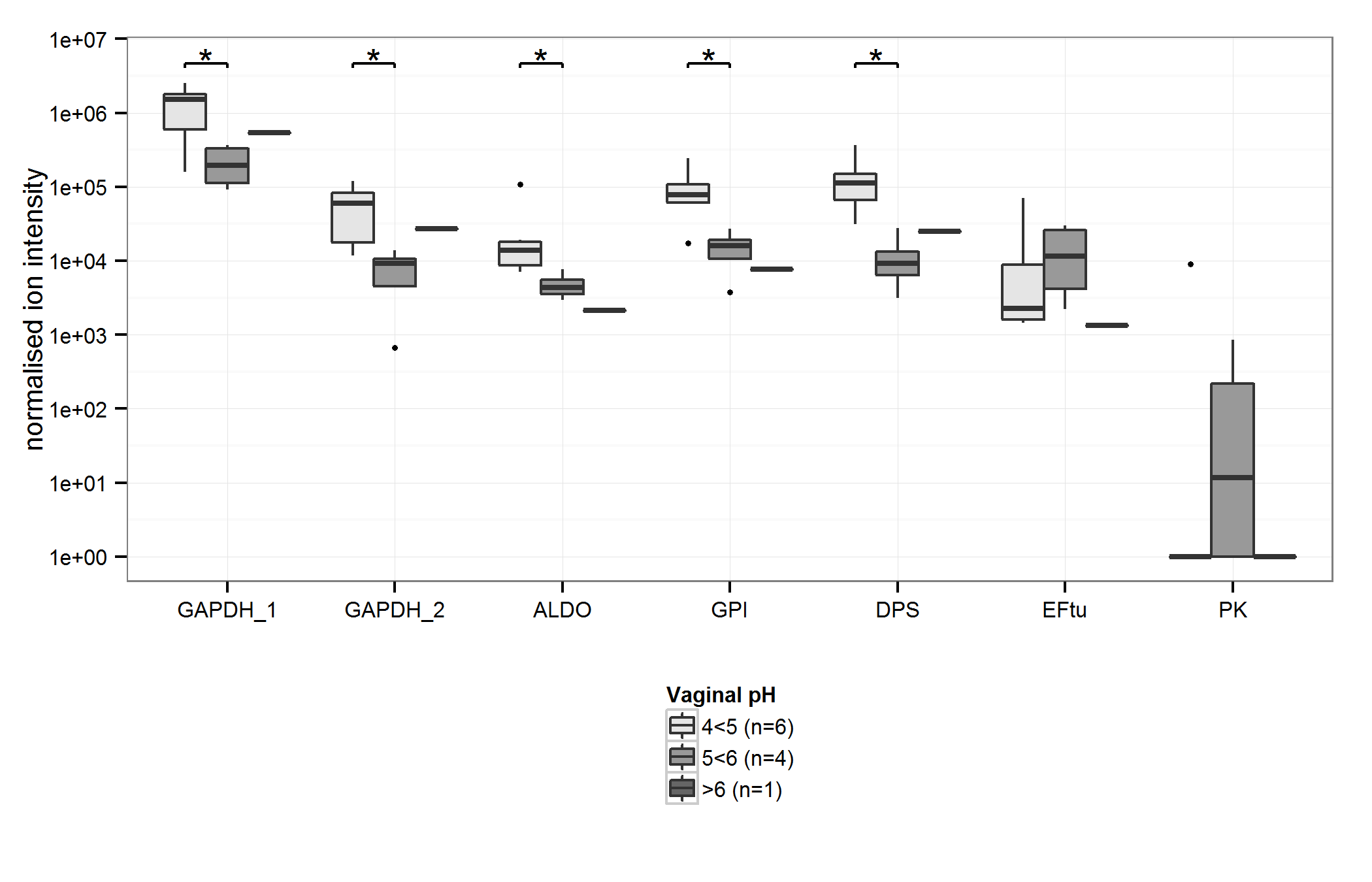

Supplement: S2 Fig — GAPDH_1, GAPDH_2, ALDO, GPI, and DPS were significantly decreased in women with a vaginal pH ≥5, compared to women with a vaginal pH between 4 and 5. Box plots represent median (black line), first and third quartiles (box) and range within 1.5 times the interquartile range from the box (whiskers). Outliers are plotted as points. *p-value<0.05. Abbreviations: GAPDH: glyceraldehyde-3-phosphate dehydrogenase; ALDO: fructose-bisphosphate aldolase; GPI: glucose-6-phosphate isomerase; DPS: DNA starvation/stationary phase protection protein; EFtu: elongation factor Tu; PK: pyruvate kinase. (TIFF) [file pone.0150767.s002.tiff]
